# Feature-selective responses in macaque visual cortex follow eye movements during natural vision

**DOI:** 10.1038/s41593-024-01631-5

**Published:** 2024-04-29

**Authors:** Will Xiao, Saloni Sharma, Gabriel Kreiman, Margaret S. Livingstone

**Affiliations:** 1 Department of Neurobiology, Harvard Medical School, Boston, MA, USA; 2 Department of Molecular and Cellular Biology, Harvard University, Cambridge, MA, USA; 3 Department of Ophthalmology, Boston Children’s Hospital, Harvard Medical School, Boston, MA, USA; 4 These authors jointly supervised this work: Gabriel Kreiman, Margaret S. Livingstone

## Abstract

In natural vision, primates actively move their eyes several times per second via saccades. It remains unclear whether, during this active looking, visual neurons exhibit classical retinotopic properties, anticipate gaze shifts or mirror the stable quality of perception, especially in complex natural scenes. Here, we let 13 monkeys freely view thousands of natural images across 4.6 million fixations, recorded 883 h of neuronal responses in six areas spanning primary visual to anterior inferior temporal cortex and analyzed spatial, temporal and featural selectivity in these responses. Face neurons tracked their receptive field contents, indicated by category-selective responses. Self-consistency analysis showed that general feature-selective responses also followed eye movements and remained gaze-dependent over seconds of viewing the same image. Computational models of feature-selective responses located retinotopic receptive fields during free viewing. We found limited evidence for feature-selective predictive remapping and no viewing-history integration. Thus, ventral visual neurons represent the world in a predominantly eye-centered reference frame during natural vision.

We see the world as stable, yet our eyes are in constant motion. How does the brain account for the movements of its visual sensor to enable stable visual perception? The question of visual stability dates back centuries to von Helmholtz, Descartes and Alhazen^1,2^. The primate ventral visual pathway, specialized in the processing of detailed visual features^3^, is a candidate for contributing to the stable perception of what is where. Ventral visual processing culminates in the inferior temporal cortex (IT) which, in two to three synapses, reaches the entorhinal cortex containing grid cells that code for spatial gaze direction^4^ and the hippocampus harboring place cells and episodic memory^5,6^, both plausibly involving gaze-independent representations.

Most studies on ventral visual neurons use passive-viewing experiments, in which images are presented in the receptive field (RF) of a neuron while the subject fixates. Some studies examining active vision found V1 and IT responses to be retinotopic^7–9^. In particular, DiCarlo and Maunsell^8^ showed that IT responses were near-identical during free and passive viewing. Other studies reported neurons that remap their spatial RFs around saccade time in ventral areas V2 (ref. 10) and V4 (refs. 11–13). Perisaccadic RF remapping, first reported in the lateral intraparietal (LIP) area^14^, is best established in the LIP, frontal eye field^15,16^ and superior colliculus^17,18^ (see reviews^19–22^). Remapping is posited to contribute to visual stability by allowing the comparison and integration of the pre- and postsaccadic scenes^1,20,22^.

Because remapping studies have probed neurons with simple transient stimuli (for example, light spots), it remains unknown whether remapped RFs transport feature information across saccades^19–21^. Moreover, stable visual perception may leverage a persistent scene rich in framing cues^19,20,22–25^. Studies have investigated feature-selective neuronal activity in primates freely viewing natural stimuli^26–31^. Interpreting these data is challenging due to the admixture of eye movements, stimulus features and selectivity in stochastic single-trial responses.

Here, we analyzed neuronal responses in six ventral visual areas in monkeys freely viewing natural images, assessing selectivity in space, in time and to stimulus features. We further tested specific hypotheses about predictive remapping and trans-saccadic integration. The results summarize 679 experimental sessions, containing 883 h of recording from 13 monkeys making 4.6 million fixations on thousands of natural images. We found that neurons throughout the ventral visual pathway selectively responded to retinotopic stimulus features, showing limited evidence for predicting future RF features or integrating the viewing history.

## Results

**Fig. 1 |:**
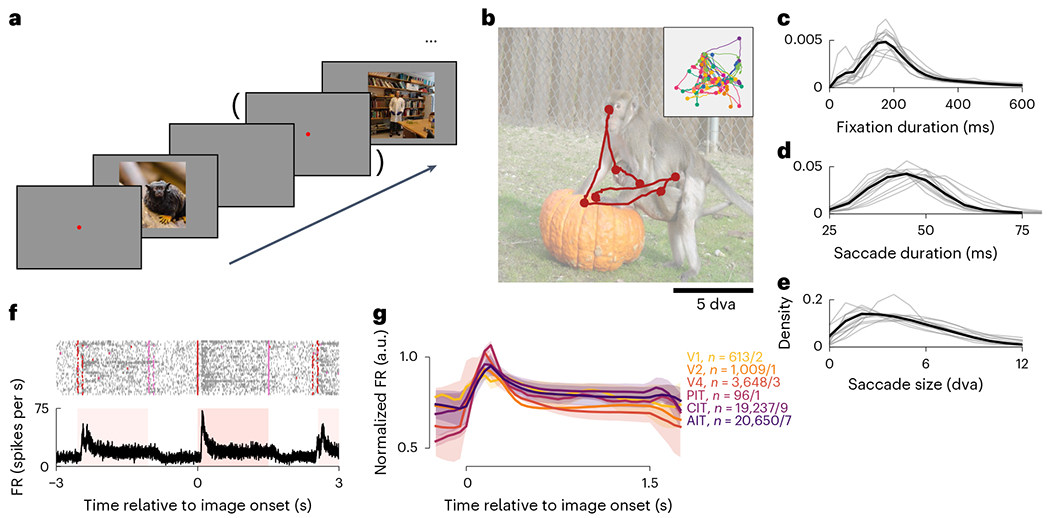
Overview ofthe free-viewing experiment. **a**, Monkeys freely viewed images presented in a random sequence. A fixation dot was displayed before some image presentations. **b**, The gaze trajectory in an example presentation. The inset shows gaze trajectories for the same image across repeat presentations in one experimental session. Colors indicate different presentations; dots, fixations. **c**-**e**, Distributions of fixation durations (**c**), saccade durations (**d**) and saccade sizes (**e**). Thin lines indicate individual monkeys; thick lines, across-monkey averages. **f**, Image-onset-aligned spike rasters and average FRs for an example AIT neuron. Red and pink ticks indicate image onset and offset times; pink-shaded regions, the typical image presentation cadence in this session. **g**, Mean normalized FRs per visual area, using presentations lasting 1.5 s for illustration. Values of *n* correspond to the number of neurons/monkeys per visual area. The shading indicates the bootstrap 95% CI of the mean.

In each session, a monkey viewed a sequence of natural images that repeated in a pseudorandom block fashion (Fig. 1a). Each image presentation lasted up to 1.5 s typically (in 410 of 679 sessions; range 0.3–60 s in other sessions) and was interrupted if the monkey looked away from the image. The images were typically shown at a size of 16 × 16 degrees of visual angle (dva; 487 of 679 sessions; range 8 × 8 to 26 × 26 dva in other sessions). Monkeys naturally looked around the images without training, examining each image with varied looking patterns across image repeats (Fig. 1b, inset). An average fixation lasted 276 ± 49 ms; an average saccade took 50 ± 5 ms and subtended 5.4 ± 0.9 dva (all mean ± s.d. across subjects; Fig. 1c–e).

We recorded extracellular single- and multi-unit activity (hereafter, neurons) using chronically implanted multielectrode arrays. The recordings spanned six visual areas: V1, V2, V4 and the posterior, central and anterior divisions of IT (PIT, CIT and AIT). Most data were collected in CIT and AIT (eight and seven monkeys; neuron and monkey numbers included are noted per plot; see the Methods for detailed inclusion criteria), followed by V4 (three monkeys), V1 (two), V2 and PIT (one each). Ventral visual neurons were generally more active during image presentations (Fig. 1f,g): mean firing rates (FRs) increased following image onset, remained elevated as the monkey explored the image and returned to baseline after image offset.

### Face-neuron responses were gaze-specific

**Fig. 2 |:**
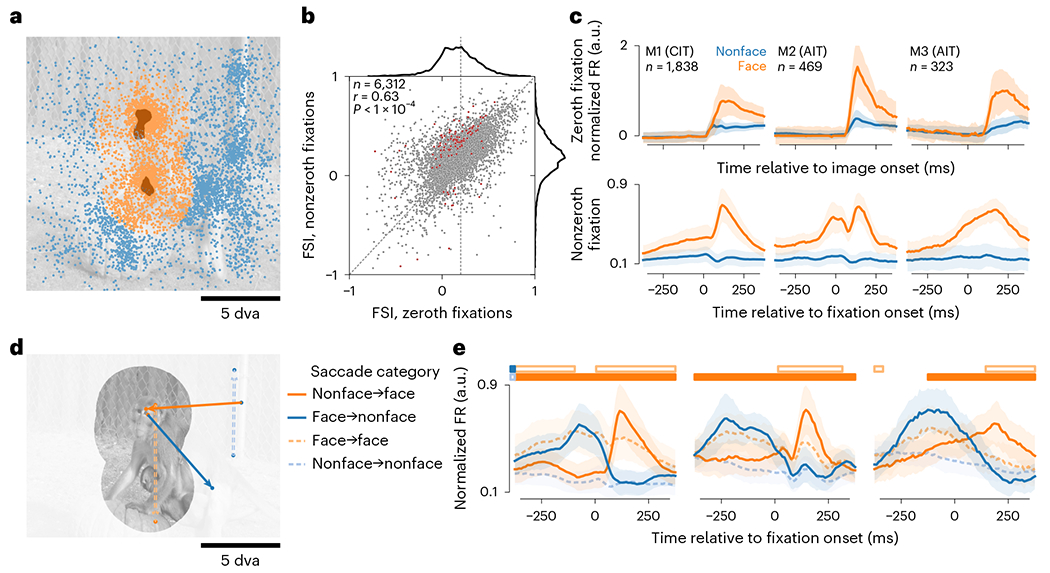
Face-selective neurons responded according to whether fixations placed RFs on a face or not. **a**, Fixations were categorized as face or nonface per neuron based on RF overlap with face ROIs, here illustrated for a foveal RF 5 dva in diameter for the same image as in Fig. 1b. The two dark-shaded areas indicate face ROIs; dots, fixations; colors, categories (orange, face; blue, nonface). **b**, Neuronal face selectivity was quantified by an index (FSI) and compared between zeroth and nonzeroth fixations (respectively, *x* and *y* axes). Each dot corresponds to a neuron. The top and right subplots show marginal distributions. Neurons colored dark red had significantly different FSI between zeroth and nonzeroth fixations (*P* < 0.01, two-tailed permutation test, FDR-corrected). The diagonal dashed line corresponds to identity; vertical dashed line, zeroth-fixation FSI = 0.2. **c**, Responses per category for face-selective neurons, aligned to image onsets (top row) or nonzeroth fixation onsets (bottom row). Each column corresponds to a monkey. The *n* indicates the number of face neurons. **d**, An example saccade is shown for each of the four categories defined by the start and end fixation categories. **e**, Responses per saccade category for the same neurons as in **c**. Horizontal bars indicate time bins where responses were significantly greater for nonface-to-face versus nonface-to-nonface saccades (lower solid bars) or face-to-face versus face-to-nonface saccades (upper open bars). In panels **c** and **e**, lines and shading indicate the median ± median absolute deviation (m.a.d.) across neurons.

To study how neuronal responses interact with eye movements and stimulus content, we first focused on face-selective neurons (face neurons, for brevity). During passive fixation, face neurons respond more to faces than nonface objects^32^. During free viewing, eye movements can bring a face into and out of a neuron’s spatial RF. Thus, we categorized fixations as face or nonface by whether the face region of interest (ROI) overlapped the RF (Fig. 2a). We recorded neurons from three face patches in three monkeys (CIT in M1 and AIT in M2 and M3). To functionally identify face neurons recorded in multielectrode arrays, we calculated a face selectivity index (FSI) using responses during the ‘zeroth fixation’, the period between the image onset and the first eye movement. In this period, the onset of a random image placed either a face or a nonface in a neuron’s RF depending on where the monkey happened to be looking. Thus, the zeroth fixation was analogous to passive viewing. We defined face neurons as those with zeroth-fixation FSI at least 0.2 (vertical dashed line in Fig. 2b), that is, at least 50% higher responses to faces than to nonfaces. Across sessions, we recorded 6,312 neurons from face-patch arrays. Of these neurons, 2,683 (42.5%) passed the FSI threshold.

Parafoveally previewing a stimulus before fixating it leads to better perception, both during reading and specifically for faces^33–35^. Therefore, we asked whether face neurons were more selective during active viewing, or ‘nonzeroth fixations’, compared with passive-viewing-like zeroth fixations. Neurons had correlated FSI in the two conditions (Fig. 2b; *r* = 0.63, *n* = 6,312, one-tailed *P* < 10^−4^; all *P* values here and below were based on permutation tests with 10,000 permutations unless noted otherwise). Few neurons had significantly different FSI between zeroth and nonzeroth fixations (Fig. 2b; 39 of 2,683 (1.5%) face neurons and 108 of 6,312 (1.7%) neurons in face-patch arrays; all statistical significance values here and below were at false discovery rate (FDR)-corrected *P* < 0.01).

**Extended Data Fig. 1|:**
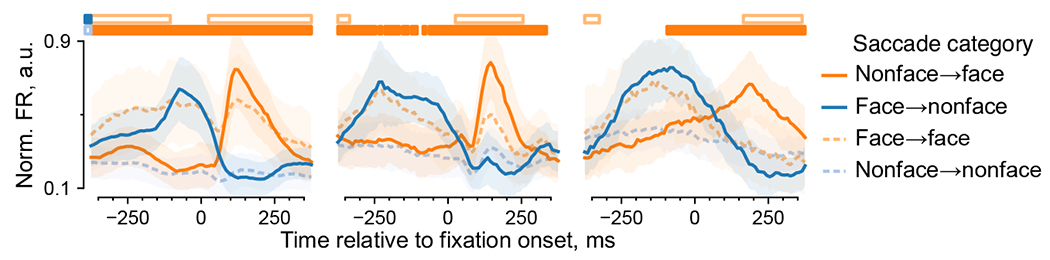
Responses per category for saccades larger than 4 dva. Each subplot corresponds to the same monkey and face neurons as in Fig. 2c, e. Lines and shading indicate the median ± median absolute deviation (m.a.d.) across neurons.

We next examined the dynamics of face-neuron activity. Face-selective responses followed image onsets (Fig. 2c, top row) and appeared to precede fixation onsets (Fig. 2c, bottom row). To account for the possibility that the apparent predictive responses arose from consecutive face fixations, we divided saccades into four categories by the start and end fixation category (Fig. 2d). Face-neuron responses followed the fixation category across saccades (Fig. 2e). For example, in nonface-to-face saccades, face-neuron activity increased around fixation onset, whereas in face-to-face saccades, responses were lower than responses following nonface-to-face saccades, consistent with response adaptation. Responses following nonface-to-face saccades were higher than nonface-to-nonface responses, and the differences became statistically significant before fixation onsets (Fig. 2e). Significant prefixation differences persisted for large (≥4 dva) saccades (Extended Data Fig. 1), indicating that presaccadic RF overlap with postsaccadic faces did not fully explain the prefixation differences. To further assess these putative predictive responses, we next sought a metric that did not require binary delineations of neuronal RFs and preferred image features.

### General feature-selective responses were gaze-specific

**Fig. 3 |:**
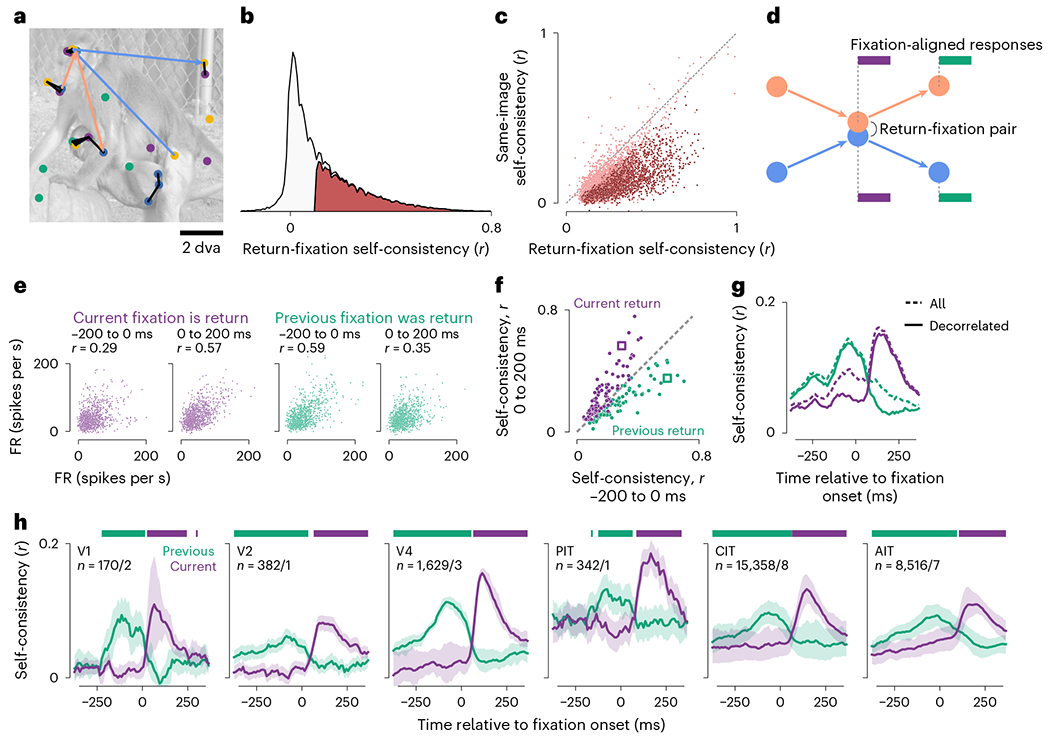
Response self-consistency during return fixations indicated gaze specificity. **a**, Example return-fixation pairs, each comprising two nearby fixations (within 1 dva) on an image within or across presentations. Dots indicate fixations; color, different presentations; black lines, return-fixation pairs; arrows, two example fixation sequences meeting in a return-fixation pair. **b**, Distribution across neurons of return-fixation self-consistency. Red indicates neurons deemed visually selective. **c**, Self-consistency per neuron between return fixations (*x* axis) or any two fixations on the same image regardless of distance (*y* axis). Each dot indicates a neuron, showing 5,000 examples; dark red, neurons with statistically significant differences between return-fixation and same-image self-consistency (*P* < 0.01, one-tailed permutation test, FDR-corrected); dashed line, identity. **d**, Schematics illustrating two rules to pair responses and calculate self-consistency. Orange and blue indicate the example fixation sequences in panel **a**; purple and green bars, responses paired based on the respective rule, ‘the current (previous) fixations are (were) return fixations’. **e**–**g**, Illustration of how we quantified self-consistency. **e**, Each dot indicates a neuron’s FRs in a return-fixation pair. The *x* and *y* axes correspond to each of the two fixations. The four subplots show responses 200 ms preceding or following fixation onsets, paired by the current or previous fixations. Because FRs were discrete and often overlapped, dots were slightly jittered for visualization purposes only. **f**, Self-consistency for all neurons in the example session. The *x* and *y* axes correspond to the two response time bins; colors, the pairing rules; each dot within a color, a neuron; square markers, the example neuron in **e**; dashed line, identity. **g**, Self-consistency for responses in 50-ms sliding bins, averaged over neurons in the example session. Dashed lines correspond to all return-fixation pairs; solid lines, decorrelated pairs. **h**, Mean decorrelated self-consistency time courses over monkeys and neurons, separately per visual area. The *n* values indicate the number of neurons/monkeys per visual area; shading, the bootstrap 95% CI of the mean; horizontal bars, time bins with significantly higher self-consistency for current- than previous-return fixations (purple) or vice versa (green; *P* < 0.01, one-tailed permutation test, FDR-corrected).

We devised a general readout for selective responses using the prevalent return fixations. Monkeys and humans repeatedly foveate parts of visual scenes above chance frequency in diverse task contexts including free viewing^36^. Figure 3a shows example return-fixation pairs (distance ≤ 1 dva) in a session, within an image presentation and between repeats. If neurons selectively respond to retinotopic features, responses should be similar between return fixations. To quantify this, we calculated response correlations between each pair of return fixations, across pairs. This measure is analogous to the self-consistency calculated between trial split halves during passive viewing, but because freely viewing monkeys can revisit each image location a different number of times, we calculated self-consistency for per-fixation (single-trial) responses.

We used self-consistency to identify neurons with robust feature selectivity. Of all 66,260 neurons across sessions, 26,975 (40.7%) had return-fixation self-consistency *r* ≥ 0.1 and significantly above zero (Fig. 3b). We focused on these neurons throughout the study because all analyses relied on feature selectivity. Although the threshold *r* = 0.1 is lower than typical values in passive-viewing studies, the single-trial activities considered here are necessarily more stochastic than standard trial-averaged responses.

To distinguish gaze specificity from overall feature selectivity, we compared response self-consistency between return fixations or any two fixations (nearby or not) on the same image (Fig. 3c). Of the 26,975 feature-selective neurons, 95.7% showed higher self-consistency during return fixations, and 60.7% reached statistical significance. Thus, almost all feature-selective neurons were specific to the gaze location.

To study the dynamics of gaze-specific responses, we calculated self-consistency for response time courses aligned to fixation onsets (Fig. 3d–h). The first two subplots in Fig. 3e illustrate the responses of an example neuron 200 ms before and after return-fixation onsets; the responses correspond to purple bars in Fig. 3d. Responses were more self-consistent after fixation onsets than before (*r* = 0.57 versus 0.29). Although the self-consistency was positive even before fixation onsets, consecutive fixations (that is, separated by one saccade) were often nearby (Fig. 1e), introducing correlations. To discern the contribution from the previous fixation, we examined responses following previous-return fixations (green in Fig. 3d,e). Comparing responses paired by previous-return fixations to those paired by current-return fixations, self-consistency was higher prefixation (Fig. 3e, third versus first subplot; *r* = 0.59 versus 0.29) and lower postfixation (second versus fourth subplots; *r* = 0.57 versus 0.35). These relations hold for most neurons in the same session (Fig. 3f).

**Fig. 4 |:**
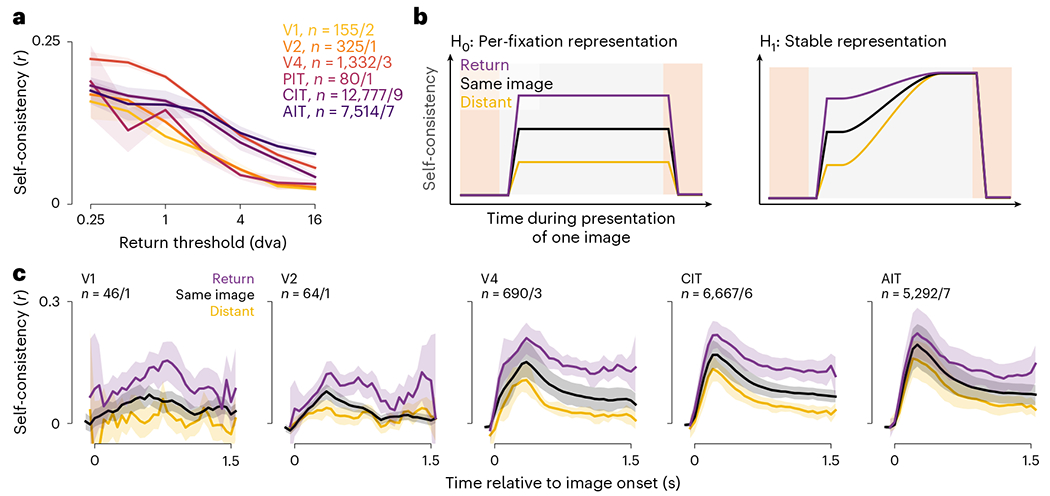
Response self-consistency showed spatial precision and no fixation integration. **a**, Self-consistency per visual area as a function of the distance threshold for defining return fixations. Lines and shading indicate the median and its bootstrap m.a.d. **b**, Schematics of predictions by the null hypothesis of per-fixation responses (H_0_, left) and the alternative hypothesis of an integrating stable representation (H_1_, right). Colors indicate response self-consistency between fixations separated by various distances: purple, return fixations ≤1 dva apart; black, fixations on the same image irrespective of distance; yellow, distant fixations >8dva apart. **c**, Mean self-consistency time courses throughout an image presentation to compare against the predictions in **b**. Lines and shading indicate the mean and its bootstrap 95% CI.

**Fig. 5 |:**
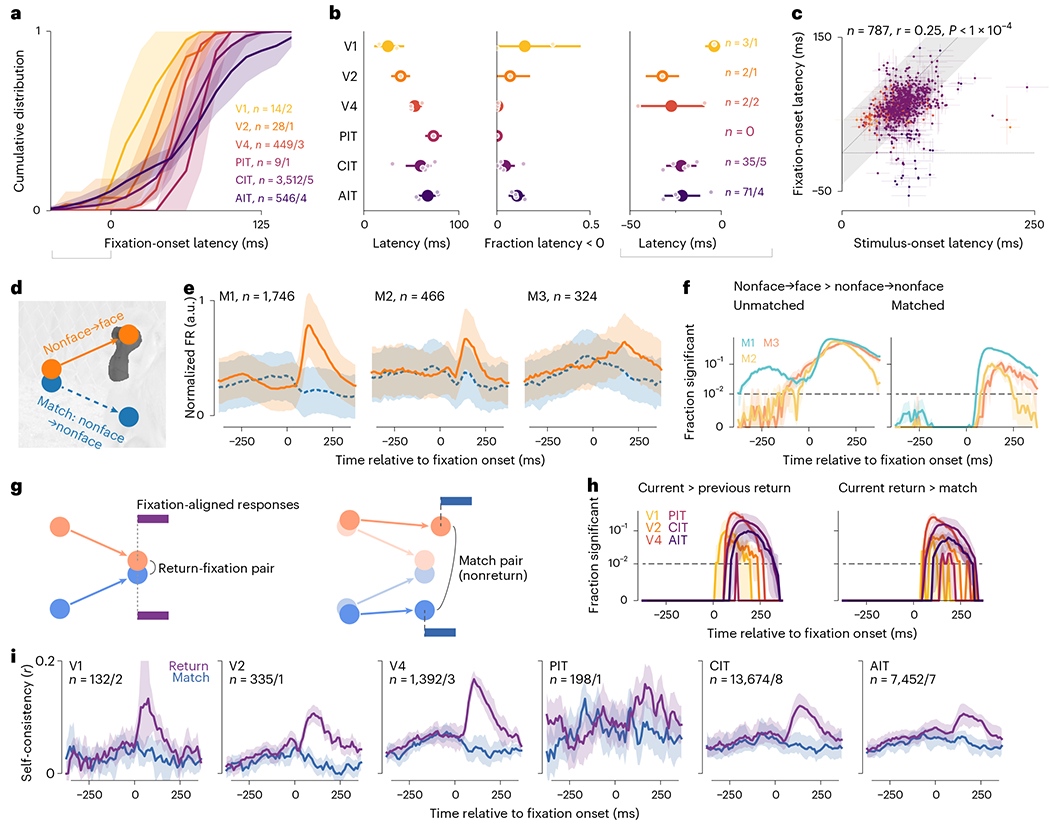
Limited evidence for predictive remapping. **a**, Mean cumulative distribution per area of response latencies following fixation onset. Shading indicates the bootstrap 95% CI of the mean; gray horizontal bracket, neurons with latency < 0 further characterized in the right two plots in panel **b**. **b**, Mean estimates per area for latency (left), the fraction of neurons with negative latencies (middle) and latency for those neurons (right; numbers indicated). Larger dots and error bars indicate overall mean ± bootstrap 95% CI; smaller dots, means per monkey. **c**, Comparison of response latency following image and fixation onsets. Each dot indicates a neuron; error bars, bootstrap s.d.; colors, visual areas as in **a** and **b**; gray shading, identity ± 25 ms; the *P* value, one-tailed permutation test. **d**, Schematics of how saccades were matched for the face-specific analysis. Each nonface-to-face saccade was matched with a nonface-to-nonface saccade that started nearby (≤1 dva) and ended far away (≥4 dva). **e**, Face-neuron responses per monkey and saccade category. Lines and shading indicate the median±m.a.d. across neurons. **f**, The fraction of neurons that responded significantly more to nonface-to-face versus nonface-to-nonface saccades for unmatched (left) and matched (right) saccades. To visualize small *P* values, the *y* axis is linear for *P* =0–0.01 and log-scaled for *P* =0.01–1. Statistical tests were per-neuron Mann-Whitney *U* tests (unpaired samples) when saccades were unmatched and Wilcoxon ranked-sum tests (paired samples) when saccades were matched (both one-tailed *P* < 0.01, FDR-corrected). Colors indicate monkeys; the shading, the bootstrap 95% CI. **g**, Schematics showing how saccades were matched for the self-consistency analysis. Individual saccades were matched as above, and we further required the match-saccade pair not to constitute a return-fixation pair. **h**, Plots showing the fraction of neurons with significantly higher self-consistency in current-return pairs than previous-return pairs (left), or current-return pairs than match pairs (right). Colors indicate visual areas. **i**, Self-consistency time courses for current-return-fixation pairs and match pairs. In panels **h** and **i**, lines and shading indicate the mean and its bootstrap 95% CI.

We evaluated the dynamics of gaze-specific responses at a higher resolution by calculating self-consistency in 50-ms sliding time bins (Fig. 3g, dashed lines). Responses to current-return fixations (purple) became more self-consistent following fixation onsets. Conversely, for previous-return fixations (green), self-consistency decreased after the (current) fixation onset. To further control for the nonpaired fixation, we excluded return-fixation pairs (current or previous) where the nonpaired fixations (preceding or following) were within 4 dva. This decorrelation procedure specifically reduced self-consistency in the nonpaired period (compare solid and dashed lines in Fig. 3g). Thus, we used the decorrelated self-consistency in subsequent analyses (Figs. 3h, 4a and 5a–c).

**Extended Data Fig. 2|:**
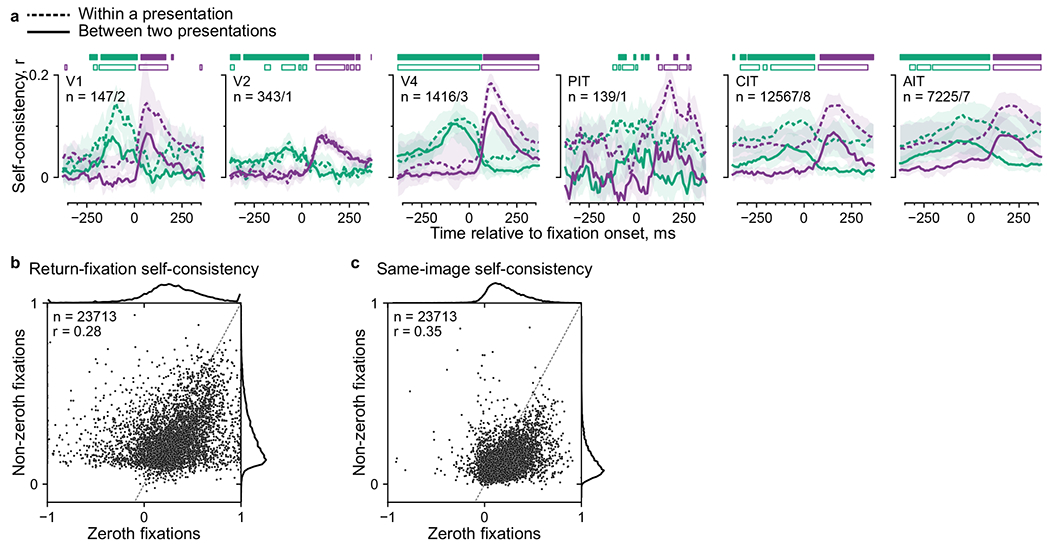
Self-consistency comparison between within- and across-presentation fixations and between zeroth and non-zeroth fixations. **a**, Return-fixation self-consistency was calculated separately for fixation pairs within a presentation or between two presentations. Lines and shading indicate the mean and its bootstrap 95% CI; horizontal bars, time bins with significantly higher self-consistency for current than previous return fixations (purple) or vice versa (green; p < 0.01, one-tailed permutation test, FDR-corrected), separately for within-presentation and between-presentation types (open and filled bars, respectively). **b, c**, Return-fixation self-consistency (**b**) and same-image self-consistency (**c**) separately for zeroth fixations (*x* axis) or non-zeroth fixations (*y*axis). Each dot indicates a neuron, showing 5,000 examples; dashed line, identity; top and right subplots, marginal distributions across neurons (numbers indicated as *n* values). The self-consistency was correlated between zeroth and nonzeroth fixations (*r* = 0.28. 0.35) and slightly lower for non-zeroth fixations (difference = 0.03, 0.08; *P* < 10^−156^, *P* ≈ 0, Wilcoxon signed-rank test, uncorrected), consistent with Fig. 4c.

Figure 3h shows the average decorrelated self-consistency time courses for visually selective neurons, separately per visual area. The responses showed gaze specificity across areas. This conclusion did not change for within- and between-presentation return fixations analyzed separately (Extended Data Fig. 2a).

### Precise spatial selectivity and no fixation integration

The self-consistency measure furnished a readout for the spatial precision of free-viewing responses. We assessed whether closer-by fixations had higher self-consistency by varying the threshold that defined return fixations. The self-consistency increased for closer-by fixations (that is, lower thresholds; Fig. 4a) down to 0.25 dva across all areas, approaching our eye-tracking resolution. Thus, ventral visual neurons had surprisingly precise spatial selectivity during free viewing.

**Extended Data Fig. 3 |:**
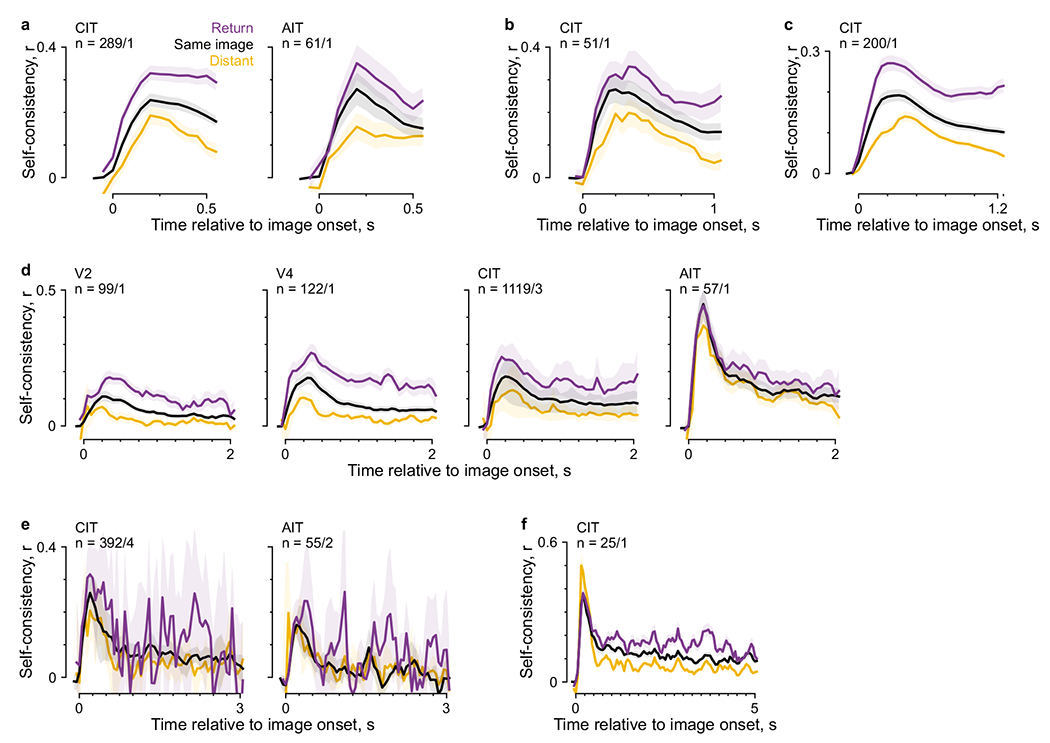
Mean self-consistency time courses throughout an image presentation for various presentation durations. Panels **a–f** relate to Fig. 4e. Each panel corresponds to a different presentation duration (indicated on the *x* axis), showing trial durations and regions with at least 25 neurons. Lines and shading indicate the mean and its bootstrap 95% CI.

Neuronal responses that reflected each gaze change are in principle compatible with integration over fixations to provide a useful stable representation^37^. If the responses integrated over fixations, as the monkey continued to view an image, increasingly similar responses should accompany different fixation locations, and the gaps should narrow (Fig. 4b, right, alternative hypothesis H_1_) among the self-consistency for return fixations ≤1 dva apart, all fixations on the same image and distant fixations >8 dva apart. In contrast, under the null hypothesis of retinotopic responses (Fig. 4b, left, H_0_), the three self-consistency measures should remain different. We tested both hypotheses for presentations lasting 1.5 s, our most common design (Fig. 4c; Extended Data Fig. 3 shows the results for other presentation times with sufficient data.) The self-consistency measures remained different throughout an image presentation, consistent with the null, retinotopic, hypothesis and contradicting the hypothesis of an integrating stable representation. However, the null hypothesis does not predict the drop in self-consistency throughout a presentation, a drop that may relate to the overall FR decrease during a presentation (Fig. 1f,g).

### Limited evidence for predictive remapping

**Extended Data Fig. 4 |:**
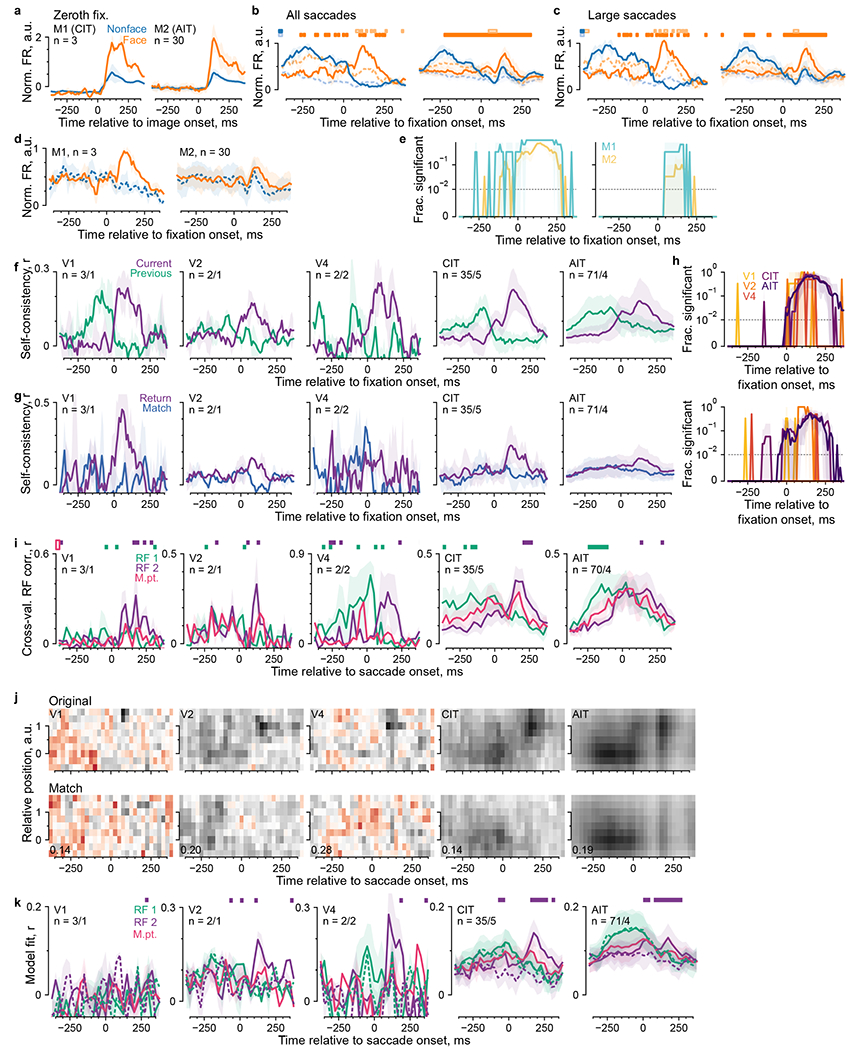
Results separately for neurons showing negative latencies relative to fixation onsets. The neurons are the same as in Fig. 5b, third subplot. Panels **a**-**k** respectively correspond to Fig. 2c (top row); 2e; Extended Data Fig. 1; Fig. 5e; f; 3h; 5i; h: 6f; 7c; and 7d. Lines and shading indicate the same center and spread estimates as in the corresponding figure panels.

**Fig. 6 |:**
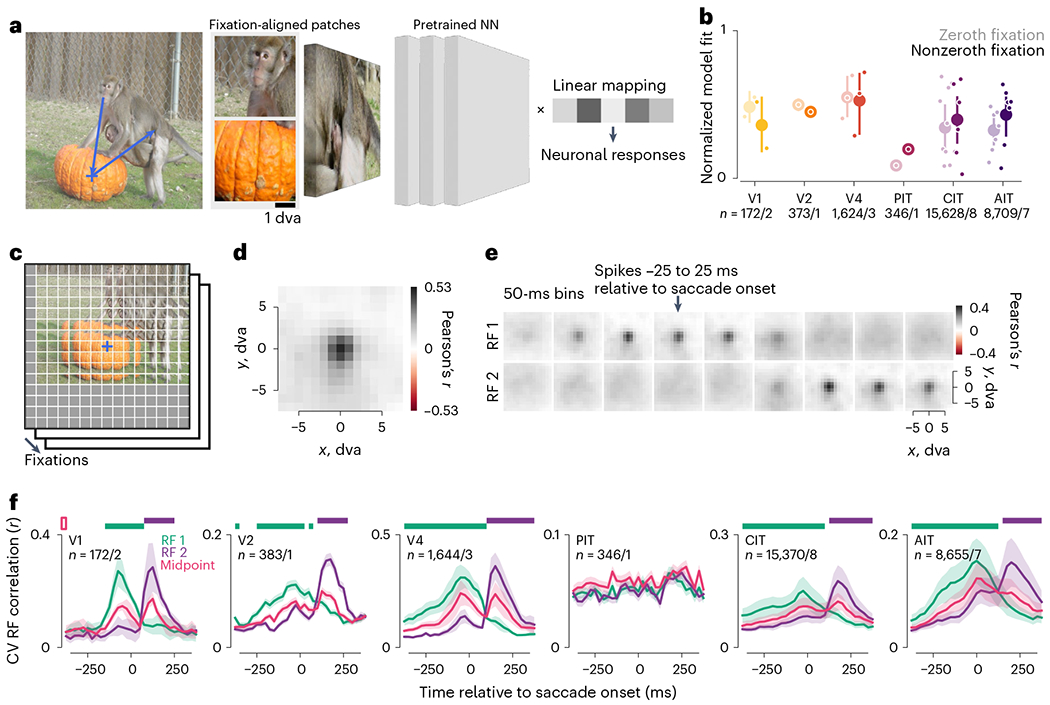
Computational models predicted per-fixation responses from stimulus features and revealed gaze-locked RF. **a**, Illustration of image-computable models for per-fixation free-viewing responses. The models comprised a pretrained, fixed neural network (NN) feature extractor and a different linear mapping fit to each neuron’s responses. Model inputs were fixation-centered image patches, shown here for an example fixation sequence (blue line and crosshairs). **b**, Normalized model fit per area for zeroth fixations and nonzeroth fixations (left and right in each pair). Larger dots and error bars indicate overall mean ± bootstrap 95% CI; smaller dots indicate means per monkey. **c**, Illustration of model-based RF mapping. The eye-centered image per fixation was partitioned into 2-dva patches on a grid of offsets centered on the fixation indicated by a cross. The NN feature extractor converted each patch into a feature vector. Model fit to neuronal responses was separately assessed at each offset from the fixation. **d**, Model-inferred RF for an example CIT neuron using fixation-onset-aligned responses. **e**, Model-inferred RFs for the same neuron using 50-ms sliding time bins aligned to saccade onsets. The arrow indicates the time bin centered on saccade onsets (−25 to 25 ms). The two rows correspond to RFs anchored to the pre- or postsaccadic fixation (RFs 1 and 2). **f**, Quantification of RF presence over time. Colors indicate RF 1 (green), RF 2 (purple) and the midpoint control (magenta); lines and shading, the mean ± bootstrap s.e.m; horizontal bars, statistically significant differences from the midpoint control (*P* < 0.01, one-tailed permutation test, FDR-corrected).

**Fig. 7 |:**
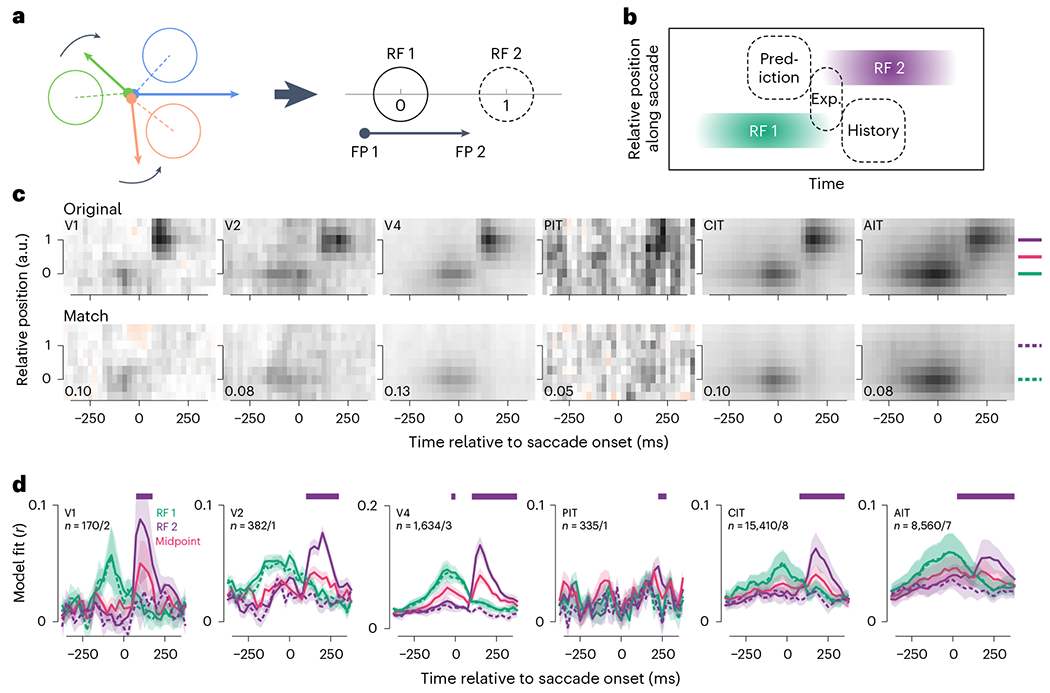
Saccade-normalized RF models showed no perisaccadic RF expansion or history integration and limited prediction. **a**, Saccade vectors (left) were aligned, rotated and scaled to a normalized vector (right) to place RFs 1 and 2 in a joint map. **b**, Schematics for how thejoint map represents retinotopic RFs and putative perisaccadic properties–predictive forward remapping (‘prediction’), perisaccadic expansion (‘exp.’) and history integration (‘history’). **c**, Model-based RF maps per area, averaged over neurons. The bottom row shows maps from models using original stimulus features but match-saccade responses to control for RF 1 contents. Both maps per area use the same value range, indicated to the lower left of the bottom plots; the color map is otherwise the same as in Fig. 6d,e. **d**, RF model fits at locations indicated by the colored lines to the right of panel **c**. Solid and dashed lines, respectively, correspond to original and match-saccade maps. The plot and associated statistical tests adjust the match-saccade model fits to correct for imperfect saccade matching (see the Methods for rationale and details). Horizontal bars indicate statistically significant differences between original and match-saccade RF 2 values (*P* <0.01, one-tailed permutation test, FDR-corrected). Lines and shading indicate the mean ± bootstrap s.e.m.

We asked whether our data provided any evidence in the ventral stream for predictively remapping neurons, which respond to stimuli in the future RF before saccade onset and may contribute to visual stability. Predictive remapping responses should have negative latencies relative to fixation onsets. The self-consistency time courses (Fig. 3h) supplied a measure for feature-selective response latency. We determined the time responses became better explained by the current fixation than the previous one, that is, the crossing point of the previous- and current-return self-consistency curves (Fig. 3g,h). The population latency distribution was mostly positive, was typical of ventral visual areas and increased along the processing hierarchy (Figs. 5a,b, left). A minority of neurons showed negative latencies (gray brackets in Fig. 5a,b). The fraction of negative-latency neurons ranged from none in PIT to 11% (6% to 15%) in AIT and 15% (0% to 45%) in V1 (mean and bootstrap 95% confidence interval (95% CI)). The negative latencies ranged from −4 ms (−9 to −1 ms) in V1 to −32 ms (−41 to −23 ms) in V2 (mean and bootstrap 95% CI; Fig. 5b, right). Because saccades took 50 ms on average (Fig. 1d) and we estimated latency relative to fixation onsets (that is, saccade offsets), these latency values do not anticipate saccade onsets, although a small number of neurons had latencies around −50 ms (for example, Fig. 5c). To cross-examine other evidence for the negative-latency neurons, we pooled all time-resolved analyses for only these neurons (Extended Data Fig. 4). Their self-consistency time courses crossed over before fixation onset, by construction (Extended Data Fig. 4e). The subset of face neurons responded early to nonface-to-face saccades (Extended Data Fig. 4a,b) as did face neurons overall (Fig. 2). RF modeling analyses, described below (Figs. 6 and 7), also allowed negative-latency neurons suggestive evidence for predictive responses (Extended Data Fig. 4c,d,f–j). Thus, a minority of neurons might respond before fixation onsets, although not before saccade onsets as in classical predictive remapping^14,20,22^.

Short of negative latency, fixation-specific responses could be faster than image-onset responses. We directly compared the fixation- and image-onset latencies in 787 neurons for which we could estimate both with bootstrap s.d. < 25 ms. The two latencies covaried across neurons (Fig. 5c; *r* = 0.27, *P* < 10^−4^). Fixation-onset latencies were statistically smaller than image-onset latencies by 19 ± 29 ms (mean ± s.d. across neurons; *P* < 10^−62^, one-tailed Wilcoxon signed-rank test), a modest population-level difference below the variance of individual estimates.

We derived latency as an indirect measure from self-consistency. To more directly test for predictive remapping, we identified ‘matched saccades’, pairs of saccades whereby a monkey started from nearby (≤1 dva) locations to acquire divergent (≥4 dva) targets (Fig. 5d). Matched saccades provided natural experiments to control, per saccade, for the presaccadic retinotopic stimulus. In the face-specific analysis, we looked for a nonface-to-nonface saccade (match) for each nonface-to-face saccade (template). Figure 5e shows category-average responses as in Fig. 2e but for matched saccades. Figure 5f shows the fraction of neurons with significantly higher responses to nonface-to-face than nonface-to-nonface saccades, separately per monkey. Without matching saccades, this fraction exceeded the chance level before fixation onsets in all monkeys (Fig. 5f, left), consistent with the population-level statistics (Fig. 2e). With matched saccades, more neurons than chance showed statistical differences only after fixation onsets (Fig. 5f, right). Thus, individual face neurons did not show significant predictive responses after accounting for the presaccadic stimulus.

Leveraging matched saccades, we devised an analogous control for the self-consistency analysis (Fig. 5g–i). For each (current) return-fixation pair, we tried to match each constituent saccade as above and further required the two match saccades not to comprise a (current) return-fixation pair. If prefixation responses contained predictive components, possibly mixed with retinotopic components, prefixation self-consistency should be higher for actual return-fixation pairs than nonreturn match pairs. Figure 5h, left, compares previous- and current-return self-consistency, showing neuron-level statistical test results to complement the population-level tests in Fig. 3h. Figure 5h, right, and 5i compare matched saccades and show that no individual neuron had significantly higher self-consistency before fixation onsets than explained by presaccadic inputs. Thus, we did not find feature-selective predictive remapping responses in individual ventral visual neurons.

### Computational models predicted per-fixation responses

The results so far showed that, during free viewing, ventral visual neurons were selective to stimulus features in space and time just as during passive viewing, encouraging us to test whether deep neural network (DNN)-based, image-computable models for passive-viewing responses^38^ could also predict free-viewing responses. We adapted these models to predict per-fixation responses from an image patch (for example, 4 × 4 dva) anchored to the fixation (Fig. 6a). A pretrained DNN (a vision transformer (ViT)^39^) converted each image patch into a feature vector. We fit a linear mapping from feature vectors to neuronal responses and evaluated predictions using cross-validation (CV) across images.

**Extended Data Fig. 5 |:**
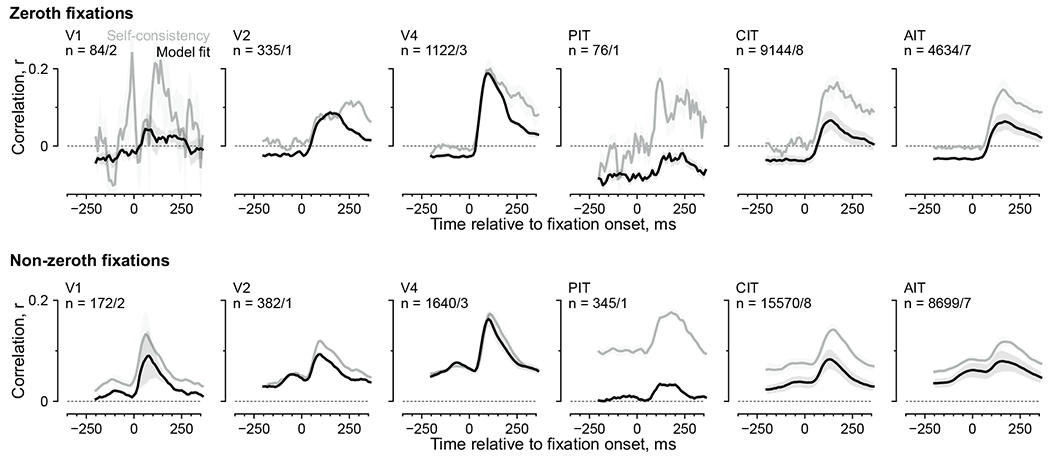
Time-resolved model fits. Mean return-fixation self-consistency (gray) and unnormalized model fit (black) time courses for zeroth fixations (first row) and non-zeroth fixations (second row), separately per visual area. Lines and shading indicate the mean ± bootstrap s.e.m.

While previous work validated similar models on trial-averaged passive-viewing responses, we found the models also predicted single-trial free-viewing responses (Fig. 6b and Extended Data Fig. 5). Models captured a similar fraction of the explainable (that is, self-consistent) responses during passive-viewing-like zeroth fixations and free viewing (nonzeroth fixations). Model fits varied across visual areas, although areas were not directly comparable due to variations in images and data size (number of fixations) and modeling choices such as the DNN layer and image-patch size.

### Models revealed retinotopic RFs

**Extended Data Fig. 6 |:**
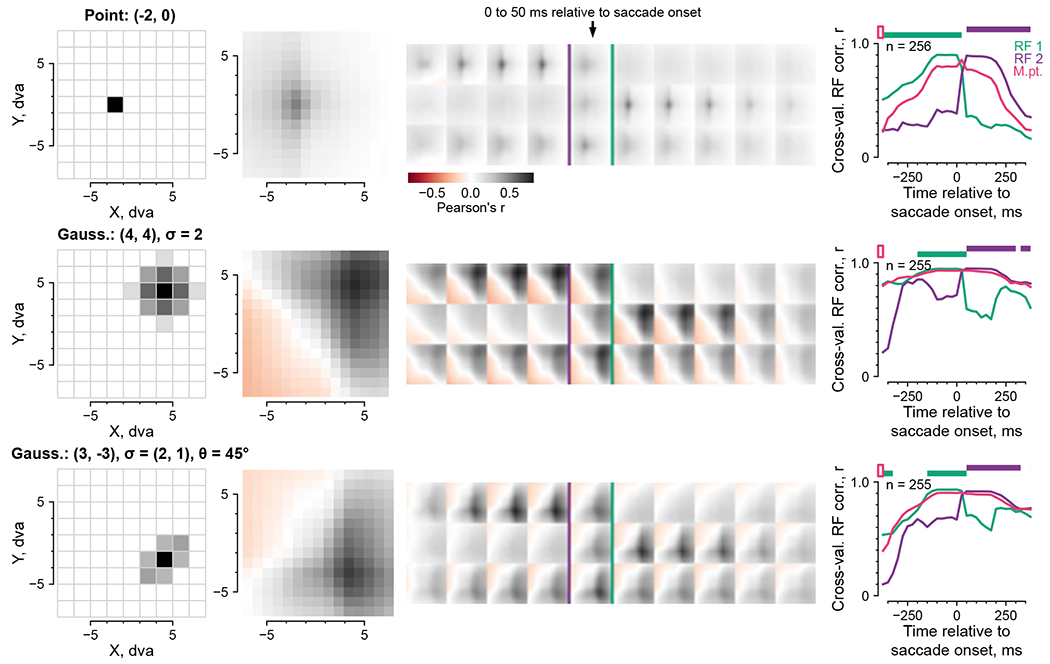
Validation of model-based RF mapping using simulated responses. We simulated retinotopic, stimulus-selective responses using behavior data from an example session. The simulated responses were a weighted spatial combination of ResNet-50 features (layer3.4.conv1, 256 channels) over 2 × 2 dva image patches centered on the eye position (with no time lag). Each channel was treated as a different neuron. Here, each row corresponds to a different simulated RF. The first column illustrates the image patches and weights used to approximate the RF. The second column shows maps for fixational periods and corresponds to the third column averaged over −150 to 0 ms (relative to saccade onsets) for RF 1 and 50 to 200 ms for RF 2. In the third column, the first two rows in each set of maps correspond to Fig. 6e but are averaged over the 256 channels. The third row corresponds to the midpoint control. The arrow indicates the 0–50 ms time bin relative to saccade onsets; the purple and green lines indicate saccade onsets and typical saccade offsets. The fourth column corresponds to Fig. 6f. Lines and shading indicate the mean ± bootstrap s.e.m.

The models provided a means to infer neuronal RFs during free viewing: models should predict a neuron’s responses using stimulus features within the RF, but not outside. To test this, we partitioned the scene centered on each fixation into a grid of 2 × 2-dva image patches at 1-dva intervals (Fig. 6c). A model used image patches at each offset from fixation to predict neuronal responses across fixations; separate models were fit on patches at different offsets. We empirically found it helpful to regularize the models by sharing linear mapping coefficients (representing a neuron’s feature selectivity) across offsets, resulting in a metric reminiscent of reverse correlation. This procedure generated a model-fit map that should correspond to a neuron’s spatial RF. Using simulated responses, we validated that this mapping procedure recovered the location and approximate size of ground-truth RFs (Extended Data Fig. 6).

**Extended Data Fig. 7 |:**
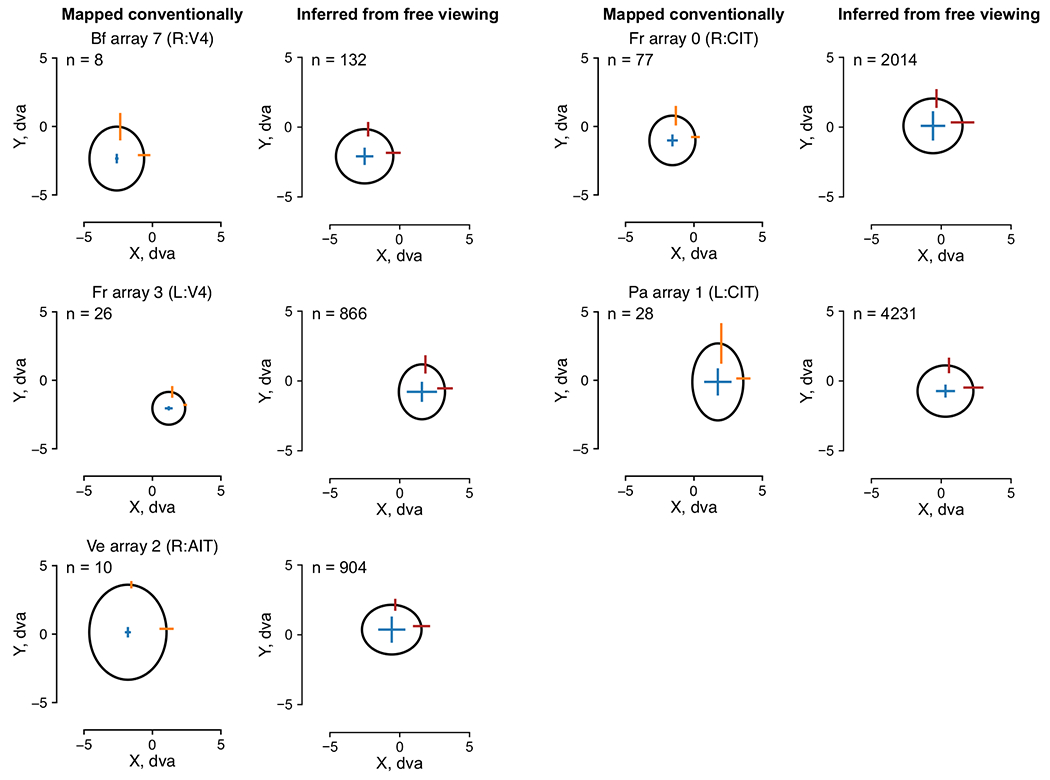
Model-inferred RFs during free viewing matched conventionally mapped RFs. RFs recovered from traditional mapping data (FRs as a function of location in response to small stimuli flashed at various locations) were compared to model-inferred RFs (Fig. 6c, d). In each subplot, the ellipse and error bars indicate the median and m.a.d. across neurons within an array of the center and size of RF Gaussian fits. The five pairs of plots show five example arrays; titles indicate the monkey, hemisphere, and visual area of the arrays. Columns 1 and 3 correspond to conventionally mapped RFs; columns 2 and 4 correspond to model-inferred RFs from free viewing data.

**Extended Data Fig. 8 |:**
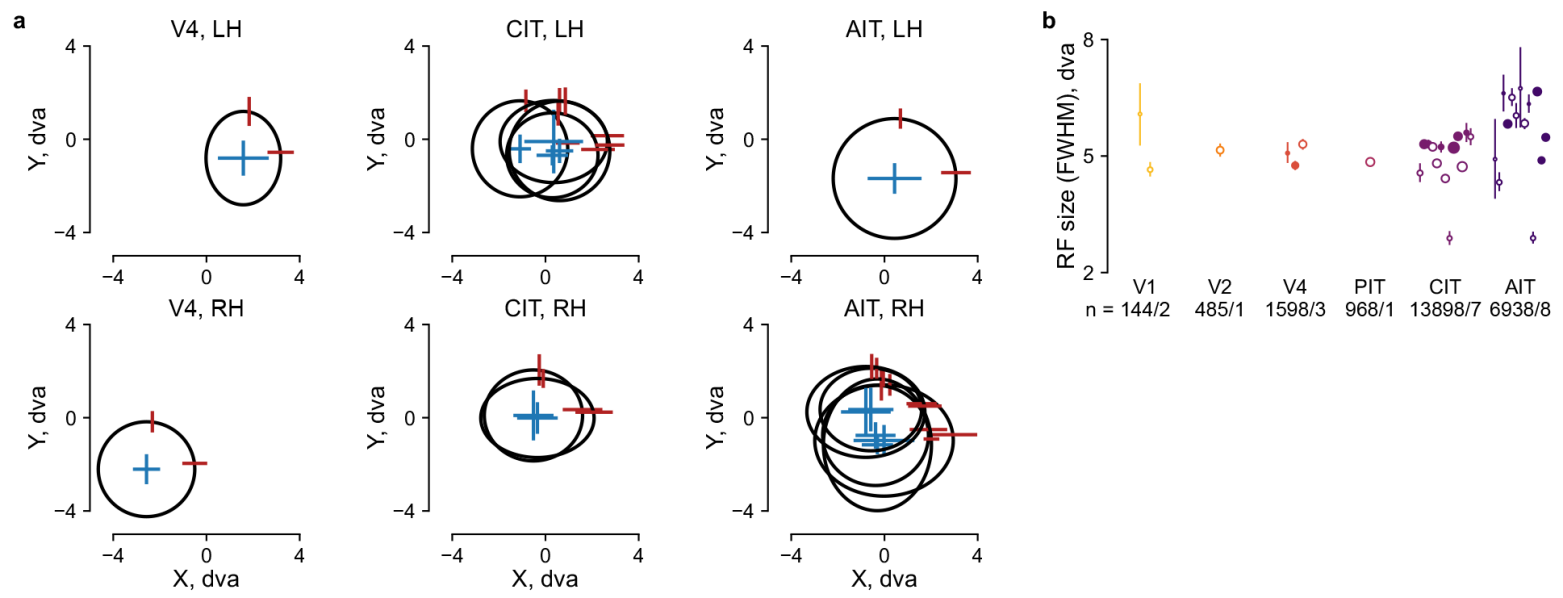
Summary of model-inferred RFs across arrays. **a**, Each subplot corresponds to a visual area and hemisphere. Each ellipse indicates the center and size of RF Gaussian fits for a chronic recording array. Error bars show the m.a.d. across neurons within an array. For clarity, only arrays with RF-center m.a.d. ≤ 1.5 dva are shown. **b**, Mean RF size per array. The RF size was quantified analogously to the full-width half-maximum (FWHM) of a circular 2D Gaussian. Specifically, FWHM = 2.355 × sqrt(*ab*), where *a* and *b* are the stdev. along the major and minor axes of the elliptical Gaussian fit. Dots and error bars indicate the mean and its bootstrap 95% CI per array; filled dots, arrays in panel **a**; dot size, the number of neurons per array.

Figure 6d shows the model-mapped RF for an example CIT neuron using fixation-onset-aligned responses. The RF contains a focal region of high model fit about 3 dva across. RFs inferred from free-viewing data were consistent with conventionally mapped RFs in this and other arrays (Extended Data Fig. 7; example neuron from Pa array 1). All well-fit RFs are summarized per array in Extended Data Fig. 8.

The model-based mapping method allowed us to directly examine remapping during natural image free viewing. We modeled responses in sliding time bins aligned to saccade onsets and used image patches anchored to the pre- or postsaccadic fixation point (FP 1 or FP 2) to map RFs in the pre- or postfixation retinotopic space (RF 1 or RF 2). Figure 6e shows the two sets of spatiotemporal RFs for the example neuron in Fig. 6d. The RFs were focal and shifted from RF 1 to RF 2 around 75–125 ms after the saccade onset, consistent with typical latencies in CIT plus an average saccade duration around 50 ms.

To summarize the RF dynamics across neurons, we quantified RF presence using its consistency over CV splits, regularized via Gaussian fits (Fig. 6f). Each time bin and retinotopic map (for example, RF 1 or RF 2) was quantified independently to allow for potential RF shifts non-parallel to the saccade^11,12,40^. Across visual areas, RF 1 was more evident before the saccade, and RF 2 was more evident after the saccade (Fig. 6f).

**Extended Data Fig. 9 |:**
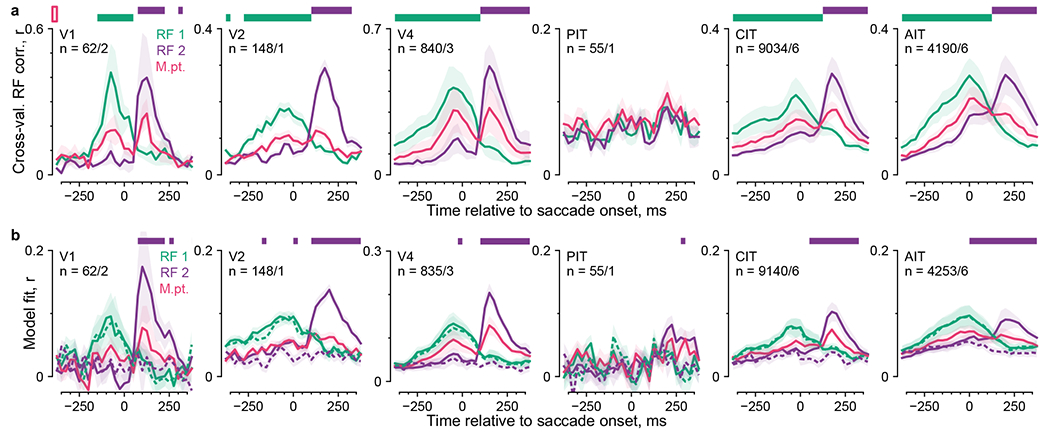
Modeling results separately for well-fit neurons. Neurons with normalized model fit of atleast 0.5 in Fig. 6b are included. Panels **a** and **b** respectively correspond to Figs. 6f and 7d. Lines and shading indicate the mean ± bootstrap s.e.m.

Similar to the self-consistency in Fig. 3h, the RF evidence was nonzero even outside the corresponding fixation period. This could indicate predictive RF remapping, memory responses or shared features between successive fixations. To distinguish these possibilities, we evaluated control RFs anchored to the midpoint of FPs 1 and 2, reasoning that the midpoint should contain common features between FPs 1 and 2. RF 2 evidence exceeded the midpoint control only after saccade onsets at the population level (Fig. 6f, top horizontal purple bars), even when we restricted the analysis to well-fit neurons (normalized model fit ≥ 0.5; Extended Data Fig. 9a). Thus, RF modeling did not indicate predictive remapping beyond retinotopic features shared across each saccade.

### Modeling showed no perisaccadic RF prediction or expansion

Figure 6 represented RFs 1 and 2 in different maps because saccade vectors varied during free viewing. To more intuitively visualize several hypotheses about perisaccadic responses, we aligned saccades by shifting, rotating and scaling them into normalized vectors such that RFs 1 and 2 were located at relative positions 0 and 1 in a joint map (Fig. 7a). Regions in this joint map readily represent three hypotheses about perisaccadic responses (Fig. 7b): predictive forward remapping^14,20,22^, perisaccadic RF expansion^41^ and viewing-history integration (Fig. 4c). RFs in the joint map were quantified using model fit. To control for the RF 1 stimulus, we again compared original (template) and match saccades analogous to Fig. 5d–i. RF maps for the original saccades revealed both RF 1 and RF 2 (Fig. 7c, top row). For match saccades, models used stimulus features along the original saccades to predict match-saccade responses, so the maps should show a weaker RF 1 (because matching was imperfect) and no RF 2. The results confirmed this expectation (Fig. 7c, bottom row).

We tested for perisaccadic expansion via the RF evidence (that is, model fit) at the midpoint between RFs 1 and 2 (relative position 0.5; Fig. 7b–d). Wang et al.^41^ showed that LIP neurons responded to midpoint stimuli at times between peak RF 1 and RF 2 responses. For neurons across ventral visual areas, the midpoint RF evidence peaked with RFs 1 and 2 (Fig. 7d), unlike LIP RF expansion and consistent with the spatial spread of classical RFs or feature similarity between the midpoint and RF contents.

The maps suggested some evidence consistent with RF 2 prediction (Fig. 7b,c, top). This evidence was not fully due to stimulus autocorrelation, which should cause symmetrical artifacts corresponding to prediction and history integration; instead, there was stronger evidence for a predictive RF 2 than an RF 1 memory (Fig. 6f and Fig. 7c, top row). Population-level statistical tests identified a time window (−50 to 0 ms to saccade onsets) in which V4 neurons had significantly higher predictive RF 2 evidence than for match saccades, even after adjusting for overall lower model fits due to imperfect matching (Fig. 7d). Considering only well-fit neurons, the V4 population still showed predictive RF 2 activity, while the V2 and AIT populations additionally showed statistically significant differences −25 to 25 ms relative to saccade onset (Extended Data Fig. 9b). Thus, modeling suggested some evidence for predictive remapping, although the putative predictive effects were modest compared with retinotopic effects (compare the solid and dashed purple lines in Fig. 7d before and after saccade onset). No similar evidence was found for history integration in any area (compare solid and dashed green lines in Fig. 7d).

## Discussion

We developed two independent analysis approaches for visual responses during natural image free viewing; these methods can benefit future studies during natural behaviors. The self-consistency measures quantify feature selectivity and its spatiotemporal specificity. The image-computable models capture feature selectivity, predict single-trial responses and map spatiotemporal RFs using natural images, extending existing methods that require simplified stimuli^9,42^. Self-consistency measures and model-based RFs corroborate each other and enable hypothesis testing about perisaccadic response properties during natural vision.

Ventral visual neurons are often approximated as retinotopic feature detectors, a classical model derived from experiments that deliberately minimize eye movements and spatiotemporal context. Studies using natural viewing conditions have hinted at nonclassical response properties^26–31^ but have not compared rigorous retinotopic models. Results here show that ventral pathway neurons retain key retinotopic properties during free viewing, although we found two deviations from strict retinotopy. First, FRs and selectivity decreased during an image presentation (Figs. 1g and 4c). Future work is needed to test whether this is explainable by adaptation mechanisms^43,44^. Second, we did not entirely rule out predictive remapping, though any predictive components are likely subsidiary to retinotopic responses. Latency estimates (Fig. 5a–c) suggest that a minority of neurons may predictively respond before fixation onset. Modeling results with population-level statistics indicate that V2, V4 and AIT may contain predictive responses (Fig. 7d and Extended Data Fig. 9b). Meanwhile, two analyses that more tightly controlled for the presaccadic stimulus did not find individual predictive neurons (Fig. 5d–i). Caution is needed with negative statistical results; future work using more data or better computational models may offer stronger evidence for predictive responses in the ventral pathway during natural vision. Our results are compatible with some neurons mixing predictive with retinotopic responses among mostly retinotopic neurons and consistent with reports that natural conditions suppress remapping through simultaneous stimuli, landmarks and background illumination^21,25,45,46^.

Indeed, viewing conditions distinguish this work from most remapping studies, which use dynamic, simple probes on an otherwise empty screen^21^. While dynamic scenes are relevant to many behaviors, much vision occurs in static environments. Further, natural scenes are feature-dense and continuously stimulate neuronal RFs, underscoring the need for remapping theories to account for feature selectivity^20,21,35^. Whether remapping transports feature information is an open question^19–21^. Our results show that the ventral visual pathway, traditionally associated with feature processing, evinces limited feature-selective predictive remapping. Static images hinder direct tests of nonpredictive or memory remapping, which has been reported in V4 (refs. 11,12,42), MST^47^, frontal eye field^16^ and superior colliculus^18,25^, and proposed to enable a transient spatiotopic representation^25,48,49^. A testable hypothesis is that memory remapping can enhance response selectivity, but we found similar face selectivity during zeroth and nonzeroth fixations (Fig. 2b) and reduced response self-consistency for nonzeroth fixations (Fig. 4c and Extended Data Fig. 2b,c). Our RF model (Figs. 6 and 7) opens future directions to assess memory remapping and transient spatiotopic integration using movie free viewing^29,31,50^.

The brain need not store a detailed, stable map of the visual world. Perception does not include a veridical image, a truism evident in idioms such as ‘out of sight, out of mind’. Vision research abounds in findings of imperfect visual stability^51^, such as inattentional (change) blindness^52^, memoryless visual search^53^ and perisaccadic mislocalization^54^. Detailed visual memory is unnecessary when we can easily re-fixate^19,36^. However, behaviors such as the multi-step saccade task^55^ show spatiotopic visual information can be preserved across eye movements. While the nature and contents of stable vision are unclear, our results suggest that the brain does not stabilize the rich representations of ventral vision.

Finally, we underscore the value of testing theories of brain function during natural behaviors. Reductionist experiments illuminate the mechanisms of cognition only insofar as the isolated facets reflect how the brain operates during normal conditions. The brain evolved for behavior, with which neuroscience should start and end^50,56^. Natural behavior is a source for generating hypotheses and should be the final test for principles gleaned from artificial experiments. We have developed flexible analyses that can be applied to study visual response properties across brain areas during natural behaviors.

## Methods

### Experiment details

#### Subjects.

All procedures were approved by the Harvard Medical School Institutional Animal Care and Use Committee (protocol number IS00001049) and conformed to National Institutes of Health guidelines provided in the Guide for the Care and Use of Laboratory Animals. Eleven adult *Macaca mulatta* (one female, ten males; 5–13 kg; 2–17 years old) and two adult male *Macaca nemestrina* (13 and 15 kg; 12 and 14 years old) were socially housed in standard quad cages on 12/12-h light/dark cycles. Detailed information per session on animal sex and age is included in the raw data available on the DANDI archive (Data availability). No statistical methods were used to predetermine sample sizes but our sample sizes are similar to those reported in previous publications (for example, refs. 8,27,42). Data collection and analysis were not performed blind to the conditions of the experiments.

#### Surgical procedures.

Animals were implanted with custom-made titanium or plastic headposts. After several weeks of fixation training, the animals underwent secondary surgeries for array implantation. All surgeries were done under full surgical anesthesia using sterile technique.

#### Physiological recording.

Animals were implanted with custom floating microelectrode arrays (32 channels, MicroProbes, or 128 channels, NeuroNexus) or microwire bundles (64 channels, MicroProbes). Each animal received 1–5 arrays throughout data collection spanning 3 years. Neural signals were amplified and sampled at 40 kHz using OmniPlex data acquisition systems (Plexon). Multi-unit spiking activity was detected using a threshold-crossing criterion. Channels containing separable waveforms were sorted online using a template-matching algorithm. The numbers of neurons were reported as sums over sessions. Neural signals were synchronized by transistor-transistor logic events to task and eye-tracking data, and synchronized image-onset event times were refined using photodiode signals. We measured and corrected for fixed lags in the eye-tracking signals as described below.

#### Behavioral task.

Monkeys performed a free-viewing task with a range of parameters, all documented in a standard format in the shared data on DANDI (Data availability). As an overview, images were typically presented at a size of 16 × 16 dva, while some experiments used other sizes ranging from 8 × 8 to 26 × 26 dva. Most experiments used a 1.5-s presentation duration, while some used other durations ranging from 0.3 to 60 s. Images were pseudorandomly ordered in a block design and repeated when all images had been shown once. In most experiments, the image position was randomly shifted in each presentation to encourage free looking, because most monkeys have been extensively trained to fixate. Monkeys were rewarded at fixed intervals with a drop of juice for maintaining their gaze within a window around the image. Task control was handled by a MATLAB-based toolbox, NIMH MonkeyLogic^57^. The task-control software monitored and recorded eye-tracking signals.

#### Eye tracking.

Monocular eye-tracking signals were acquired at 1 kHz from infrared eye trackers (ISCAN or EyeLink) without digital smoothing or filtering. Analog outputs from ISCAN trackers were sampled at a higher rate (1 kHz) than the camera frame rates (60 Hz, 120 Hz and 240 Hz, respectively, for three rigs), while the EyeLink 1000 camera sampled at a native 1-kHz frame rate. Because any tracking signal delay would lead to apparent predictive responses in the analyses, we empirically measured the end-to-end lag with a mechanized model eye rotating on a crankshaft. Eye-tracking signals were compared with signals from a potentiometer attached to the crankshaft to measure lag. The trackers had consistent delays of 46 ± 0.2 ms (60 Hz ISCAN; mean ± s.d. over 1.5-s signal segments), 37 ± 1.3 ms (120 Hz ISCAN), 24 ± 1.2 ms (240 Hz ISCAN) and 5 ± 0.8 ms (EyeLink 1000). The manufacturer specification is 0.25-dva resolution for the EyeLink 1000 system and 0.5 dva for the ISCAN systems. We did not independently measure the tracking precision. We calibrated the tracking using a projective transform before each session and during sessions as needed.

### Quantification and statistical analysis

#### Fixation detection and selection.

Fixations and saccades were detected offline using ClusterFix^58^ with default parameters. ClusterFix clips outliers (3 s.d.) and downsamples the tracking signals to 30 Hz to improve the signal-to-noise ratio. ClusterFix uses *k*-means clustering on four parameters (distance, velocity, acceleration and angular velocity) to detect fixations. A fixation was included in the analyses if it lasted at least 100 ms and landed in the image.

#### Neuron selection.

During preprocessing, we removed units with no spikes in the second half of each session or mean FRs more than 50% different between the first and second half of each session because these units were likely artifacts. All analyses except those in Figs. 1g and 3b concerned the subset of visually selective neurons based on self-consistency criteria *r* ≥ 0.1 and *P* < 0.01, as described in the main text. To ensure meaningful comparisons across time and conditions, each analysis (plot and associated statistics) only included neurons with valid values in all time bins and conditions; invalid values resulted when there were too few return-fixation pairs (at least two are needed to calculate correlations) or when FRs did not vary across the fixations qualifying for an analysis (variations are needed to calculate normalized FRs and correlations). Finally, because we calculated statistics weighing monkeys equally (see Center estimates and statistical tests), monkeys with few neurons in a region contributed noisy estimates that disproportionately affected the population averages. Therefore, we excluded, plot-by-plot, monkeys that contributed fewer than 5% of the median neuron number across monkeys per region. This criterion affected only 0–0.2% of neurons.

#### Per-neuron parameter estimates (latency and RF).

We analyzed fixation-aligned responses in a fixed time window in Figs. 2b, 3b,c and 5b. To select the time window, we estimated the response latency per neuron using the crossing point between two return-fixation self-consistency time courses, as described in Figs. 3d–g and 4a–c and further below. We selected reliable latency estimates using the conservative criteria of bootstrap s.d. < 25 ms and no other crossing points within 100 ms of the latency. The estimates for most neurons (94.3%) did not meet these strict criteria (also compare *n* values in Figs. 5a and 3h). Thus, we also considered responses pooled over neurons first per electrode, then per array. We imputed missing values using results from first the same electrode (4.1%), then the same array in each session (20.0%) and finally the same array across sessions (51.4%). For the remaining 19.8% of neurons, we set the latency to a default value per region (40, 40, 50, 65, 80 and 100 ms, respectively, for V1, V2, V4, PIT, CIT and AIT). The latency was lower-bounded at 40 ms. Imputed, default and clipped values are not reported as results (Fig. 5a–c).

We used neuronal RF locations in the analyses in Figs. 2, 5d–f, 6b and 7. The RF per neuron was estimated based on a Gaussian fit to the model-based RF described in Fig. 6d and further below. We only included reliable RF estimates that had peak unnormalized model performance *r* ≥ 0.2, goodness-of-fit *r* ≥ 0.7 and coverage (that is, definite integral of the Gaussian fit within the mapping window of −7 to 7 dva) ≥ 0.5. As with latency estimates, we imputed the 63.9% missing values from first the same electrode (8.9%), then the same array in each session (21.4%) and finally the same array across sessions (12.7%). For the remaining 21.0% of neurons, we used by default a foveal RF with a radius (s.d.) of 2 dva because most arrays had RFs near the fovea (Extended Data Fig. 8).

#### Face-specific analysis.

Face ROIs were either manually drawn for datasets containing both monkey and human faces or detected as bounding boxes using a pretrained face-detection neural network (RetinaFace^59^) for datasets containing human faces only. Fixations were classified as face fixations per neuron by whether a fixation landed within 1 s.d. of the neuronal RF center. To more closely match the face and nonface conditions, we considered only nonface fixations on images containing faces. The FSI was calculated using responses in the 150-ms window following the latency of each neuron. FSI was calculated as (*a* – *b*)/(*a* + *b*), where *a* and *b* correspond to face and nonface fixation responses, respectively. FSI calculation excluded face-to-face saccades to avoid response adaptation effects. Response time courses were normalized for each face neuron by the minimum and maximum FRs over time across the four saccade categories (Fig. 2d,e).

#### Return-fixation self-consistency.

The main elements of the return-fixation self-consistency have been described in the main text (Fig. 3). We calculated self-consistency using per-fixation (nonaveraged) responses aligned to fixation onsets. Figure 3b,c used responses in the 200-ms window following the latency of each neuron. Figure 4c used responses in 200-ms bins with 50-ms steps. Other self-consistency time courses used 50-ms time bins with 25-ms steps. In Fig. 4c, two response time bins were paired if any two fixations overlapping the bins satisfied the pairing rule (return, same image or distant).

#### Response latency estimates.

The fixation-onset response latency was estimated as the nearest time point to a central time (default is zero, the fixation-onset time) that the current-return self-consistency exceeded the previous-return self-consistency, both using decorrelated pairs. To assess the uncertainty in the estimates, we obtained 200 bootstrap samples of each underlying self-consistency time course by sampling with replacement the return-fixation pairs. We selected low-variance estimates based on half of the bootstrap samples and reported the standard deviation across the unused samples in Fig. 5c. To regularize the process, we started by estimating latency for array-averaged responses (32 or 64 electrodes, depending on array type). If we could estimate a latency and it had no other crossing points within 100 ms on either side, we used this latency as the central time for further estimates in this array. In the same way, we proceeded hierarchically down the levels of banks (32 electrodes), electrodes (one or more sorted units) and, finally, units. This hierarchical procedure is independent across bootstrap samples.

To estimate the image-onset response latency, we also used self-consistency instead of the more traditional average FRs to be more comparable to the fixation-onset latency. Here, we calculated self-consistency time courses using zeroth fixations only. The latency was estimated as the time self-consistency rose above half of the peak self-consistency, again nearest a central time that was zero by default. We assessed the uncertainties and hierarchically set the central time as above.

#### Computational model of neuronal responses.

The computational models comprised a pretrained (‘task-optimized’) DNN, which extracts a vector representation of image features, and a linear mapping fit to the responses per neuron, following previous work (for example, ref. 60). We used a pretrained ViT^39^ (specifically, the model instance ‘vit_large_patch16_384’ in the Python library ‘pytorch image models’^61^) and extracted features from the layer ‘blocks.13.attn.qkv’ (relative depth 0.55). The features were averaged over the sequence dimension into a 3,072-dimensional vector.

We chose the DNN model architecture and layer and the regularization hyperparameter for Ridge regression based on a grid search over 18 architectures ranging from 8 layers (AlexNet^62^) to 437 layers (EfficientNet-L2 Noisy Student, 475 × 475 resolution (ref. 63)) and trainable parameter number from 8 million (DenseNet-121 (ref. 64)) to 480 million (EfficientNet-L2, 475 × 475); over the layers per architecture; and over the Ridge regularization parameter (1 to 10^6^ in half-decade steps). The parameter search included at least one session per recording array (26 of 679 sessions; 2,379 units). The layer ‘blocks.13.attn.qkv’ (relative depth 0.55) in the model ViT-L/16 384 × 384 performed the best overall, reaching 90–100% of the performance of the best-fitting layer separately for each visual area. Due to the small performance gap, we elected to use the same layer to model all visual areas.

For computational efficiency, we precalculated and cached the DNN image representations on a discrete sampling grid, either 4 × 4-dva patches in 1-dva steps for fixation-centered models (Fig. 6a,b), or 2 × 2-dva patches in 0.5-dva steps for inferring RFs (Figs. 6c–e and 7). Patches extending beyond the image were padded with gray. Fixation locations were indexed to the closest image patch to obtain the corresponding feature vectors. Next, a Ridge regression model (regularization parameter alpha = 10^5^) was fitted between model features and neuronal responses. The linear mappings were fitted and evaluated using fivefold CV across images. Thus, no return fixations straddled the training and testing sets. Model performance was quantified by the correlation (Pearson’s *r*) between predicted and actual responses on held-out fixations. Ceiling-normalized model performance (Fig. 6b) was calculated by dividing model performance with return-fixation self-consistency (excluding any values ≤0), clipping the result between 0 and 1, then squaring it. This measure, standard in the literature, corresponded to the fraction of variance explained up to an optimal linear transformation.

#### Model-based inference of RF structure.

We inferred RFs for fixation-aligned responses in the 200-ms window following the latency of each neuron (Fig. 6c,d). At each fixation, a 15 × 15 array of image patches (each 2 × 2 dva) was extracted on a fixation-anchored grid of offset locations from −7 to 7 dva in 1-dva steps. Each image patch corresponded to a 3,072-dimensional model embedding vector, resulting in a 15 × 15 × 3,072 retinotopic stimulus representation analogous to a multichannel image. At each of the offset locations, we fitted and evaluated a separate linear mapping using CV over images. This process resulted in a 15 × 15 × 5 map of model performance per CV split.

To further regularize this map, we took the model weights from the location of peak performance and applied the model to held-out fixations, projecting the 15 × 15 × 3,072 stimulus representation per fixation to a 15 × 15 scalar map per neuron. The map was akin to a grayscale image reflecting the selectivity of each neuron in each fixation-centered scene. This map was also specific to each response window and CV split. These scalar preference maps were correlated with neuronal responses in a calculation analogous to reverse correlation to result in an RF map.

To fit Gaussian RF models, we first clipped the maps at 0 because negative correlations indicated over-fitting, then squared them because doing so resulted in better Gaussian fits empirically.

To quantify the consistent presence of RFs (Fig. 6f), we fitted a Gaussian distribution to the inferred RF per CV split and then evaluated the goodness-of-fit (Pearson’s *r*) on maps from other splits. The goodness-of-fit was averaged over 5 × 4 pairs of splits (the pairs were directional because each split in turn contributed to the Gaussian fit).

For RFs across saccades (Fig. 6e,f), the above process was repeated for two retinotopic spaces anchored to the fixation point either before or after the saccade. As controls, a third set of RFs was calculated anchored to saccade midpoints. Neuronal responses were aligned to saccade onsets in 50-ms bins from −375 to 375 ms in 25-ms steps. Each time bin was modeled separately.

RFs along normalized saccades (Fig. 7) were mapped at relative positions along saccades from −0.5 to 1.5 in 0.25 steps. Each relative position for each saccade was converted to the actual position on the image to index the corresponding patch. Then, a map of model performance was calculated as above.

#### Adjusting model fit in match saccades.

Because matching saccades at the 1-dva threshold did not perfectly reproduce the retinotopic stimulation during the original saccades, model fit was slightly lower when using stimulus features along the original saccades to predict match-saccade responses even in RF 1 (Fig. 7c). We attempted a first-order correction of this drop in model performance by linearly fitting the match-saccade RF 1 time course to the original-saccade one (that is, the two conditions that would ideally be perfectly matched). To use the simplest model, we fit linear coefficients on grand average time courses over all neurons across areas. The linear fit over 31 time points achieved *R*^2^ = 0.9984 and a relative deviation of only 1.4 ± 1.1% (mean ± s.d. over time points), confirming that this heuristic adjustment was reasonable. Thus, we applied the same transformation to the RF 2 time course and performed statistical tests on the adjusted values.

#### Simulation of responses representing ground-truth RFs.

Each simulated RF was discretized into one or more offset locations in 2-dva steps, to be indexed into corresponding 2 × 2 image patches aligned to each eye position sample. Offset locations were assigned weights based on a Gaussian decay profile truncated at 2σ. Responses were simulated for each eye position sample at the native 1-kHz rate, although downstream analysis would bin responses into 50-ms time bins. A simulated response sample was the weighted sum of the model representations of image patches comprising the RF. No stochasticity was added. The simulated responses were entered into the same analysis pipeline as described above for real data. To prevent trivial fitting by the ViT model, the responses were simulated using ResNet-50 (ref. 65) embeddings at the layer ‘layer3.4.conv1’. The model implementation and ImageNet-pretrained weights were from the Python library ‘torchvision’.

#### Center estimates and statistical tests.

Center estimates (mean or median) were taken first over neurons per monkey, then over monkeys. The type of spread reported was specified in each case. In Fig. 2c,e, the spread (median absolute deviation) indicated the variation across neurons. In other figures, the spread indicated uncertainty (for example, 95% CI or s.e.m.) in the center estimate by bootstrapping first over monkeys, then over neurons per monkey 1,000 times.

We used nonparametric statistical tests without normality assumptions. Tests at the neuron level were performed separately per neuron per session, and we reported the (FDR-corrected) fraction of neurons reaching statistical significance. Because signals from chronically implanted arrays were related across days, population-level statistical tests always averaged the test statistics (real or permuted) first over neurons, then over monkeys, each weighted equally. In tests involving self-consistency, the unit of permutation was fixation pairs (for example, return fixations). In other tests, the unit of permutation was the values (for example, model fits). All permutation-based statistical tests used 10,000 permutations. *P* values were corrected per analysis to control the FDR at 0.01 using the two-stage Benjamini–Krieger–Yekutieli procedure.

## Extended Data

## Data Availability

All data necessary to interpret, verify and extend the research in this study are freely available at the DANDI archive (https://dandiarchive.org/dandiset/000628) and OSF (https://osf.io/sde8m/).
