# Supplementary material for: Feature-selective responses in macaque visual cortex follow eye movements during natural vision
Source: Nat Neurosci. 2024 Apr 29;27(6):1157–66. doi: 10.1038/s41593-024-01631-5 (PMC11156562; doi:10.1038/s41593-024-01631-5)
Supplement: Supplementary file 1 [file 41593_2024_1631_MOESM1_ESM.pdf]

Corresponding author(s): Will Xiao
Last updated by author(s): Mar 14, 2024

Reporting Summary

Nature Portfolio wishes to improve the reproducibility of the work that we publish. This form provides structure for consistency and transparency in reporting. For further information on Nature Portfolio policies, see our Editorial Policies and the Editorial Policy Checklist.

Statistics

For all statistical analyses, confirm that the following items are present in the figure legend, table legend, main text, or Methods section.

n/a	Confirmed	
☐	☒	The exact sample size (*n*) for each experimental group/condition, given as a discrete number and unit of measurement
☐	☒	A statement on whether measurements were taken from distinct samples or whether the same sample was measured repeatedly
☐	☒	The statistical test(s) used AND whether they are one- or two-sided<br>*Only common tests should be described solely by name; describe more complex techniques in the Methods section.*
☐	☒	A description of all covariates tested
☐	☒	A description of any assumptions or corrections, such as tests of normality and adjustment for multiple comparisons
☐	☒	A full description of the statistical parameters including central tendency (e.g. means) or other basic estimates (e.g. regression coefficient) AND variation (e.g. standard deviation) or associated estimates of uncertainty (e.g. confidence intervals)
☐	☒	For null hypothesis testing, the test statistic (e.g. *F*, *t*, *r*) with confidence intervals, effect sizes, degrees of freedom and *P* value noted<br>*Give P values as exact values whenever suitable.*
☒	☐	For Bayesian analysis, information on the choice of priors and Markov chain Monte Carlo settings
☐	☒	For hierarchical and complex designs, identification of the appropriate level for tests and full reporting of outcomes
☐	☒	Estimates of effect sizes (e.g. Cohen's *d*, Pearson's *r*), indicating how they were calculated

Our web collection on statistics for biologists contains articles on many of the points above.

Software and code

Policy information about availability of computer code

Data collection	Data were collected with Omniplex software (releases v1.17, v1.19, v1.20) by Plexon, Dallas, TX and NIMH MonkeyLogic software (ten versions ranging from build 187 (May 19, 2019) to version 2.2.20 (Oct 27, 2021); the detailed version per experimental session is noted in the published code repository).
Data analysis	Data were analyzed using custom Python (version 3.10) code. The code to reproduce the analyses in this paper is shared at https://github.com/willwx/free_viewing.

For manuscripts utilizing custom algorithms or software that are central to the research but not yet described in published literature, software must be made available to editors and reviewers. We strongly encourage code deposition in a community repository (e.g. GitHub). See the Nature Portfolio guidelines for submitting code & software for further information.

Data

Policy information about availability of data

All manuscripts must include a data availability statement. This statement should provide the following information, where applicable:

- Accession codes, unique identifiers, or web links for publicly available datasets
- A description of any restrictions on data availability
- For clinical datasets or third party data, please ensure that the statement adheres to our policy

All data necessary to interpret, verify, and extend the research in this study are freely available on the DANDI archive (https://dandiarchive.org/dandiset/000628) and OSF (https://osf.io/sde8m/).

Research involving human participants, their data, or biological material

Policy information about studies with human participants or human data. See also policy information about sex, gender (identity/presentation), and sexual orientation and race, ethnicity and racism.

Reporting on sex and gender	No human participants were involved.
Reporting on race, ethnicity, or other socially relevant groupings	N/A
Population characteristics	N/A
Recruitment	N/A
Ethics oversight	N/A

Note that full information on the approval of the study protocol must also be provided in the manuscript.

Field-specific reporting

Please select the one below that is the best fit for your research. If you are not sure, read the appropriate sections before making your selection.

☒ Life sciences ☐ Behavioural & social sciences ☐ Ecological, evolutionary & environmental sciences

For a reference copy of the document with all sections, see nature.com/documents/nr-reporting-summary-flat.pdf

Life sciences study design

All studies must disclose on these points even when the disclosure is negative.

Sample size

Sample sizes were not predetermined. We retrospectively analyzed free viewing sessions collected in the lab up to 9/21/2022. The sample size meet or exceeded the two-monkey standard in the field (e.g., refs. 8, 27) for most brain areas (V1, V4, CIT, AIT), while results from the other two brain areas (V2, PIT) follow the same overall conclusions. Sample sizes are clearly indicated in each analysis.

Data exclusions

No experimental sessions were excluded from the analyses. Neurons and fixations were selected using criteria described in detail in the main text and Methods.

We selected neurons by feature selectivity because we used chronic arrays that did not always record visual signals on every electrode, and all analyses (except the overviews in Figs. 1g and 3b) relied on feature selectivity. The exclusion criteria were not pre-established but were minimal and pertinent to the study questions.

We further selected neurons on a per-analysis basis for the following reasons. First, to ensure meaningful comparisons across time and conditions, each analysis (plot and associated statistics) only included neurons with valid values in all time bins and conditions; invalid values resulted when there were too few return-fixation pairs (at least two are needed to calculate correlations) or when firing rates did not vary across the fixations qualifying for an analysis (variations are needed to calculate normalized FRs and correlations). Second, because we calculated statistics weighing monkeys equally (see Center estimates and statistical tests), monkeys with few neurons in a region contributed noisy estimates that disproportionately affected the population averages. Therefore, we excluded, plot-by-plot, monkeys that contributed fewer than 5% of the median neuron number across monkeys per region. This criterion only affected 0–0.2% of neurons.

We selected fixations using minimal criteria (having a minimum duration and being within the image) pertinent to the study questions.

Replication

Independent experiments were replicated for 679 sessions across 13 monkeys; each monkey performed at most one session per day.

Randomization

Randomization was not relevant because the study described natural behaviors and responses and involved no experimental manipulations.

Blinding | Blinding was not relevant because there was no experimental manipulation or group assignment.

Reporting for specific materials, systems and methods

We require information from authors about some types of materials, experimental systems and methods used in many studies. Here, indicate whether each material, system or method listed is relevant to your study. If you are not sure if a list item applies to your research, read the appropriate section before selecting a response.

Materials & experimental systems

n/a	Involved in the study
☒	☐ Antibodies
☒	☐ Eukaryotic cell lines
☒	☐ Palaeontology and archaeology
☐	☒ Animals and other organisms
☒	☐ Clinical data
☒	☐ Dual use research of concern
☒	☐ Plants

Methods

n/a	Involved in the study
☒	☐ ChIP-seq
☒	☐ Flow cytometry
☒	☐ MRI-based neuroimaging

Animals and other research organisms

Policy information about studies involving animals; ARRIVE guidelines recommended for reporting animal research, and Sex and Gender in Research

Laboratory animals	Eleven adult Macaca mulatta (one female, ten males; 5–13 kg; 2–17 years old) and two adult male Macaca nemestrina (13 and 15 kg; 12 and 14 years old) were involved. Detailed information per session on animal species and age (the experiments spanned years for some animals) is included in the raw data available on the DANDI archive (see Data Availability).
Wild animals	The study did not involve wild animals.
Reporting on sex	The study design did not consider sex, nor were analyses segregated by sex. We did not perform sex-based analyses because our research question (what the basic principles are of visual neuronal responses during naturalistic viewing) did not focus on sex. Because 12 of our 13 animals were male, were there to be a sex-based difference, our findings will apply to male animals. Detailed information per session on animal sex is included in the raw data available on the DANDI archive (see Data Availability).
Field-collected samples	The study did not involve field-collected samples.
Ethics oversight	All procedures were approved by the Harvard Medical School Institutional Animal Care and Use Committee (protocol number IS00001049) and conformed to NIH guidelines provided in the Guide for the Care and Use of Laboratory Animals.

Note that full information on the approval of the study protocol must also be provided in the manuscript.
